# Morphometric analysis of the lumbar vertebrae and intervertebral discs in relation to abdominal aorta: CT-based study

**DOI:** 10.1007/s00276-021-02865-9

**Published:** 2021-12-07

**Authors:** Anna Kot, Jarosław Polak, Tomasz Klepinowski, Maciej J. Frączek, Roger M. Krzyżewski, Anna Grochowska, Tadeusz J. Popiela, Borys M. Kwinta

**Affiliations:** 1 Department of Orthopedics, Traumatology, Microsurgery and Hand Surgery, Specialist Hospital, Jasło, Poland; 2 Department of Neurosurgery and Neurotraumatology, Jagiellonian University Medical College, Kraków, Poland; 3 Department of Neurosurgery, Pomeranian Medical University, Szczecin, Poland; 4 Faculty of Medicine, Jagiellonian University Medical College, Św. Anny Street 12, 31-008 Kraków, Poland; 5 Department of Radiology, Jagiellonian University Medical College, Kraków, Poland

**Keywords:** Lumbar vertebrae, Aorta, Intervertebral disc, Vascular system injuries, Discectomy, Radiology

## Abstract

**Purpose:**

Although lumbar discectomy is the most common procedure in spine surgery, reports about anatomical relations between discs and prevertebral vessels are limited. Aim of this research was to investigate morphometric of the lumbar region and the relations between intervertebral discs (IVDs) and abdominal aorta.

**Methods:**

557 abdominal computed tomography scans were assessed. For each spinal column level from Th12/L1 down to L4/L5, we investigated: intervertebral disc’s and vertebra’s height, width, length, and distance from aorta or common iliac artery (CIA). Those arteries were also measured in two dimensions and classified based on location.

**Results:**

54.58% of patients were male. There was a significant difference in arterial-disc distances (ADDs) between genders at the levels: L1/L2 (1.32 ± 1.97 vs. 0.96 ± 1.78 mm; *p* = 0.0194), L2/L3 (1.97 ± 2.16 vs. 1.15 ± 2.01 mm; *p* < 0.0001), L3/L4 (2.54 ± 2.78 vs. 1.71 ± 2.61 mm; *p* = 0.0012), also for both CIAs (left CIA 3.64 ± 3.63 vs. 2.6 ± 3.06 mm; *p* = 0.0004 and right CIA: 7.96 ± 5.06 vs. 5.8 ± 4.57 mm; *p* < 0.001)—those ADDs were higher in men at all levels. The length and width of IVD increased alongside with disc level with the maximum at L4/L5.

**Conclusion:**

Bifurcations of the aorta in most cases occurred at the L4 level. Collected data suggest that at the highest lumbar levels, there is a greater possibility to cause injury of the aorta due to its close anatomical relationship with discs. Females have limited, in comparison to males, ADD at L1/L2, L2/L3, and L3/L4 levels what should be taken into consideration during preoperative planning of surgical intervention.

## Introduction

In spine procedures, posterior lumbar disc surgery is the most performed. It is considered a safe and effective treatment of herniated discs; nevertheless, the possibility of vascular injury is still considered as a potential and serious complication of lumbar spine surgery [3, 4, 10, 11, 14, 17, 20, 22–24]. One of the most dangerous and usually fatal early complications is retroperitoneal haemorrhage resulting in haemorrhagic shock [10, 22, 24]. Furthermore, the diagnosis of late complications including arteriovenous fistula or pseudoaneurysm can be established even years after the operation when the patients may develop high-output heart failure or pulmonary hypertension [14, 20, 23, 27]. The location of iatrogenic laceration is most commonly placed at the L4-L5 level [3, 22]. Reported in recent literature reviews of vascular injuries during lumbar disc surgery shown overall mortality rate varying from 18.8 to 44% [1, 3]. Although lumbar discectomy is a common procedure in spine surgery, and at the same time, it can result in serious complications; reports about anatomical relations between discs or vertebrae and prevertebral vessels, especially the aorta and common iliac arteries, are limited [4, 11, 17].

## Materials and methods

**Fig. 1:**
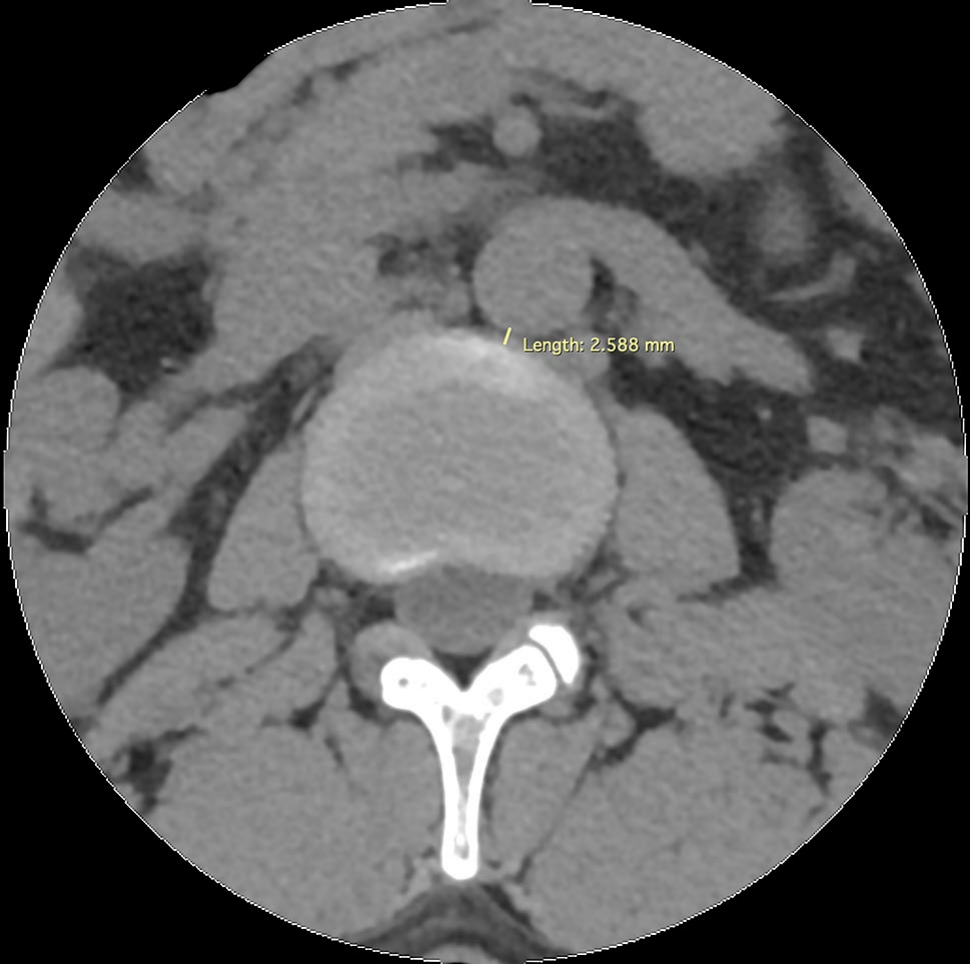
Lumbar spine computed tomography (axial) measurement of arterial-disc distance

**Fig. 2:**
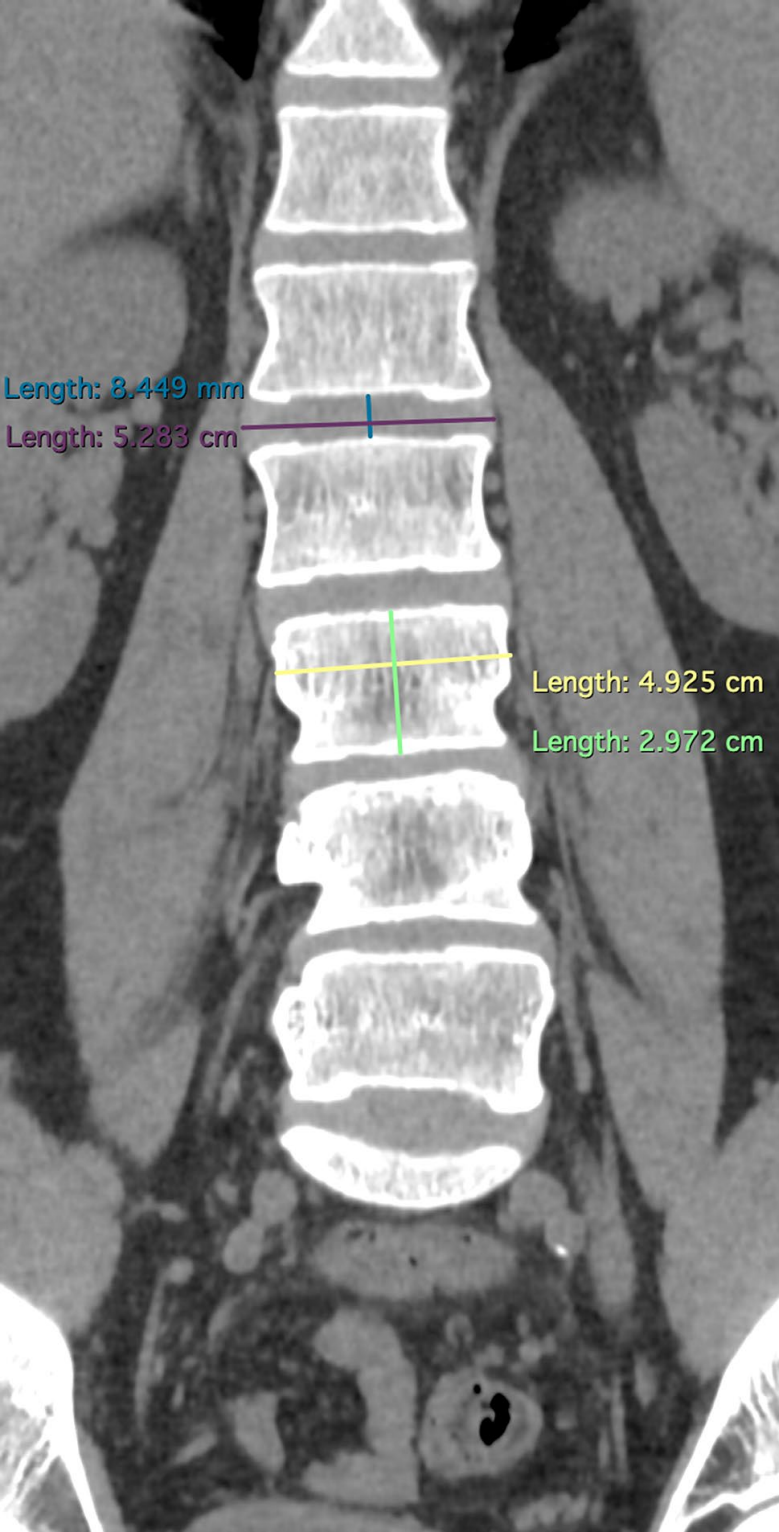
Lumbar spine computed tomography (coronal) of verterbra’s and disc’s height and width

**Fig. 3:**
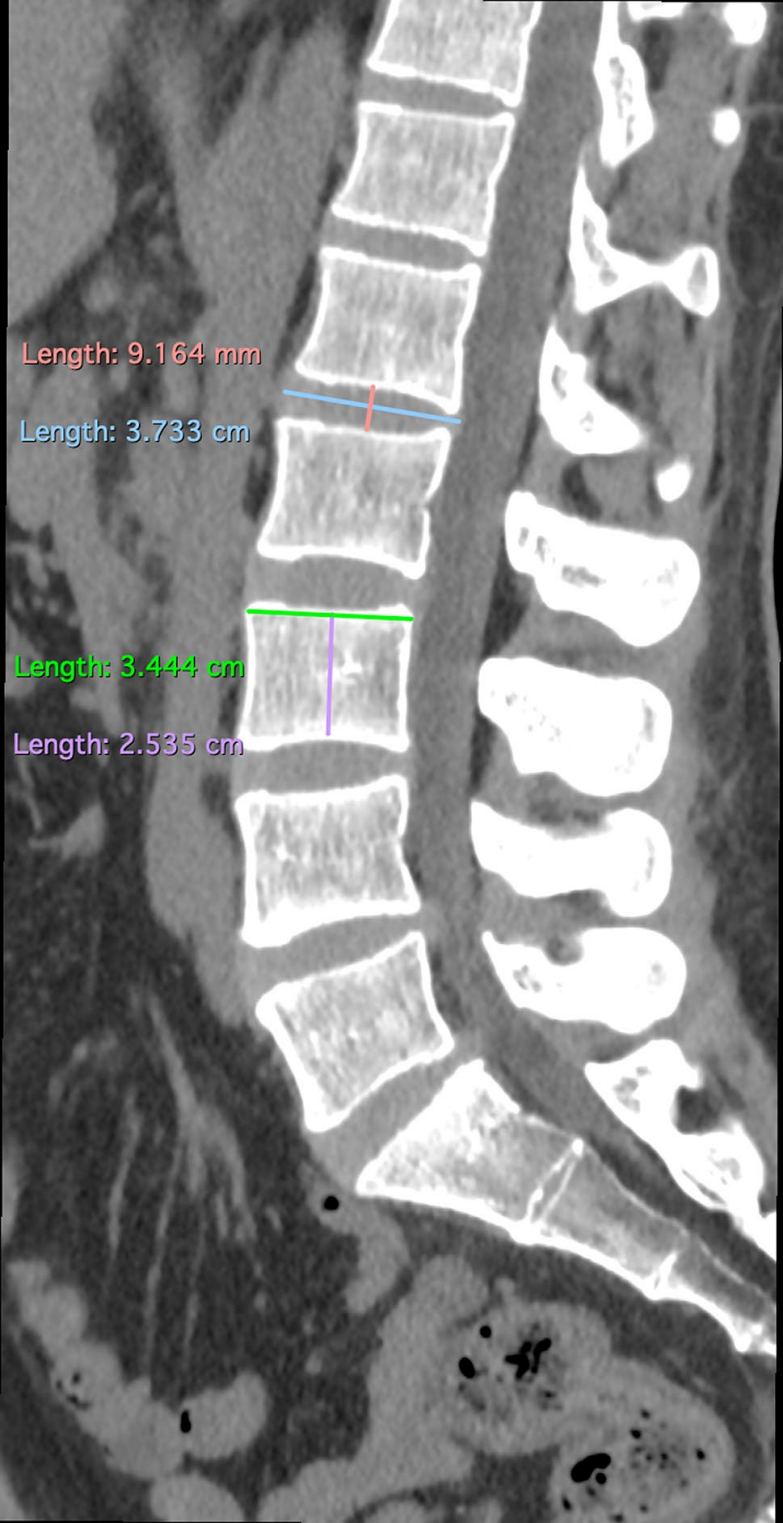
Lumbar spine computed tomography (sagittal) measurements of vertebra’s and disc’s height and length

**Fig. 4:**
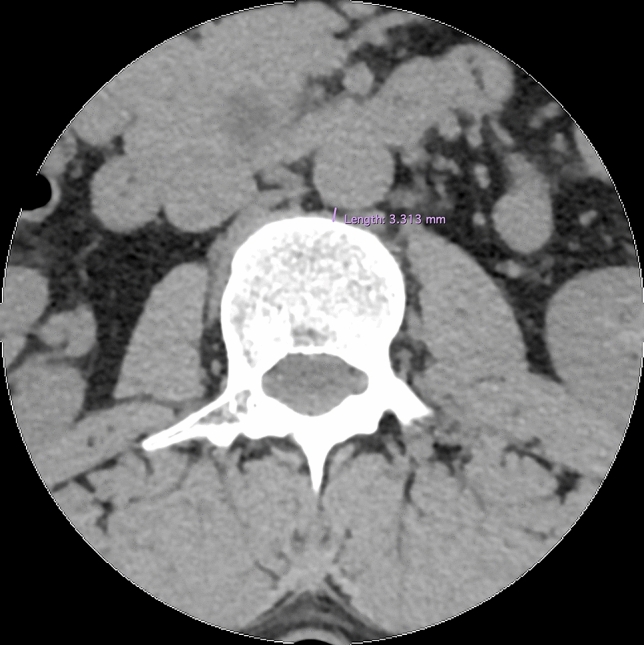
Lumbar spine computed tomography (axial) measurement of arterial-vertebra distance

**Fig. 5:**
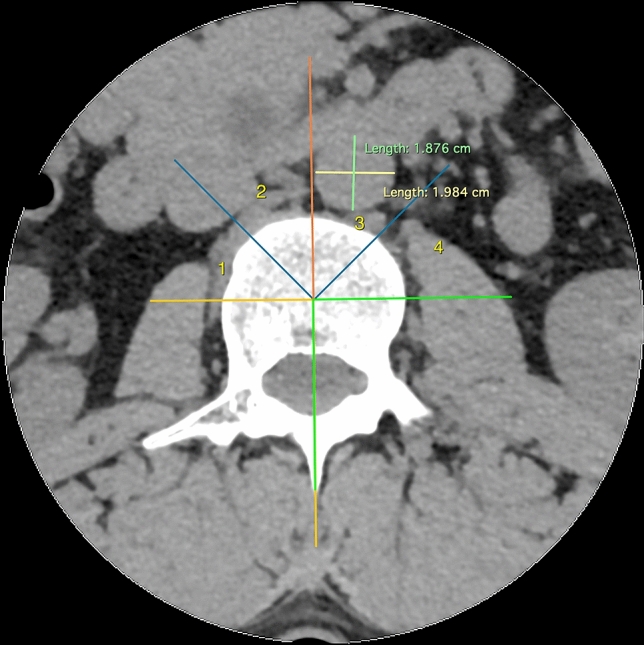
Lumbar spine computed tomography (axial) classification of the course of arterial vessels in 4 areas (1, 2, 3, 4) according to their relation to the spinal column and their measurement (sagittal and coronal). Each area is a ½ subdivision of a 90° angle from coronal plane (45° each area)

The population was selected from the patients undergoing radiological examination over 5 months (July–December 2016) during off-hours in the Department of Radiology, Jagiellonian University Medical College (Kraków, Poland). Scans were taken using 64-row computed tomography (CT) (Scanner GE Optima CT 660; GE Healthcare, Chicago, IL, USA). The scanner setting was: 120 kV, 200 mA, 64 × 0.625 mm slice collimation. Axial 0.625 mm slices at an increment of 1.25 mm were reconstructed with a matrix of 512 × 512, applying a standard kernel. Images of patients with incomplete examinations—not focusing on the vertebral column or aorta from Th12/L1 down to L4/L5 were excluded before achieving digital CT scans. 585 consecutive abdominal computed tomography scans of the patients were anonymized and achieved in an aforementioned period of time and hospital. Measurements of the disc and abdominal aorta were taken by means of RadiAnt DICOM Viewer 3.4.2 software. Measurements for each scan were taken by at least one member of group of 3 evaluators. If more members assessed scan, arithmetic mean was taken into account. Examinations for aorta malformations, such as aneurysms or aortic dissection, or great deformities of the spine, were excluded. 557 scans of patients were acquired, with a slight predominance of males (54.58%). Careful examination of scans and measurements were obtained for all patients at all disc levels (in ½ of the height of disc) and at all vertebrae levels (at the level of the upper and lower margin of the vertebrae, separately) from Th12/L1 down to L4/L5. The measurements concerning intra-vertebral discs (IVD) included: disc height, width, length, and its distance from the aorta (or common iliac artery)—arterial-disc distance (ADD) (Figs. 1, 2, 3). Those concerning vertebrae were vertebrae height, width, length, and its distance from the aorta (or common iliac artery)—arterial-vertebra distance (AVD) (for the upper and lower margin of the vertebrae separately) (Figs. 2, 3, 4). Aorta and common iliac arteries were measured at all levels (including disc and separate—upper and lower vertebrae levels) in two dimensions (sagittal and coronal). Arterial vessels courses were classified in 4 types (1, 2, 3, 4) based on their relation to the spinal column (Fig. 5). First, the center of the body of vertebrae was found in axial dimension CT (two perpendicular lines dividing the body of vertebrae in half were drawn). 4 equal angles (45 grades each), which shared sides and a common endpoint—the center of the vertebra’s body was found. The angles established 4 main, equal areas [from right to the left side from 1 (A1) to 4 (A4)]. If the vessel had a central course [half in area 2 (A2) and the other half in area 3 (A3)], it was classified to the additional category: 2½ (A2½) as there was no accessible way to category. Results were analyzed and categorized as an entire group and divided into groups according to gender.

## Results

The study group consisted of consecutive 557 patients which underwent abdominal CT examinations: 304 males and 253 females (mean age ± SD = 55.81 ± 17.77). The measurements concerning the morphometric of the spinal column are presented in Table 1. Height of the intervertebral discs increased from Th12/L1 to L3/L4 level [mean maximal height (mm) ± SD 9.53 ± 2.35]. The mean height of IVD at the L4/L5 level was smaller than at L3/L4. The length and width of IVD increased alongside disc levels with the maximum at the L4/L5 level [mean maximal length (mm) ± SD 40.57 ± 5.15; mean maximal width (mm) ± SD 59.37 ± 5.5]. Analysis of the vertebrae parameters revealed that the mean maximal height of vertebrae was at L2, and the mean minimal height was at L5 level for all patients as well as for separate male and female groups [mean maximal height (mm) ± SD 26.09 ± 2.52; mean minimal height (mm) ± SD 25.04 ± 2.66]. Mean minimal length and width of the vertebrae were at the level of the upper L1 margin [mean minimal length (mm) ± SD 33.89 ± 3.74; mean minimal width (mm) ± SD 46 ± 5.69]. Mean maximal length was at the level of the upper L4 margin and width at the level of upper L5 [mean maximal length (mm) ± SD 37.59 ± 4.26; mean maximal width (mm) ± SD 56.82 ± 5.87]. The measurements were larger in the male group considering all IVD and vertebrae-related parameters.

**Table 1:** The measurements of intervertebral disc and upper or lower margin of vertebrae considering height, length and width at the particular level of spine

	All group			Male group			Female group		
	Height [mean (mm) ± SD]	Length [mean (mm) ± SD]	Width [mean (mm) ± SD]	Height [mean (mm) ± SD]	Length [mean (mm) ± SD]	Width [mean (mm) ± SD]	Height [mean (mm) ± SD]	Length [mean (mm) ± SD]	Width [mean (mm) ± SD]
TH12/L1	6.58 ± 1.81	34.87 ± 4	47.96 ± 5.23	6.83 ± 1.66	36.75 ± 3.61	50.49 ± 4.77	6.28 ± 1.94	32.61 ± 3.20	44.93 ± 4.02
L1 U	25.48 ± 3.00	33.89 ± 3.74	46 ± 5.69	26.17 ± 3	35.88 ± 3.07	48.73 ± 5.48	24.53 ± 3.08	31.51 ± 3.01	42.73 ± 3.93
L1 L		34.58 ± 4.34	49.65 ± 5.31		36.64 ± 3.78	52.64 ± 4.4		32.11 ± 3.63	46.07 ± 3.9
L1/L2	7.75 ± 2.31	37.4 ± 4.58	52.04 ± 5.3	8.07 ± 2.13	39.52 ± 4.19	54.63 ± 4.48	7.27 ± 2.13	34.87 ± 3.66	48.96 ± 4.49
L2 U	26.09 ± 2.52	35.69 ± 4.19	49.25 ± 5.43	26.74 ± 2.55	37.85 ± 3.45	52.14 ± 4.63	25.39 ± 2.49	33.12 ± 3.49	45.8 ± 4.16
L2 L		35.99 ± 4.12	51.75 ± 6.10		37.91 ± 3.5	54.73 ± 5.95		33.69 ± 3.57	48.18 ± 4.03
L2/L3	8.96 ± 2.35	39.62 ± 5.14	55.04 ± 6.10	9.34 ± 2.43	41.49 ± 4.07	57.64 ± 5.88	8.5 ± 2.16	37.38 ± 5.38	51.93 ± 4.78
L3 U	25.99 ± 2.61	37.04 ± 4.41	51.88 ± 6	26.55 ± 2.44	39.17 ± 3.75	54.93 ± 5.4	25.32 ± 2.63	34.48 ± 3.73	48.22 ± 4.42
L3 L		36.69 ± 4.58	54.33 ± 5.69		38.72 ± 4.38	57.13 ± 4.98		34.25 ± 3.48	50.97 ± 4.55
L3/L4	9.53 ± 2.35	40.14 ± 4.74	57.77 ± 5.67	9.88 ± 2.42	42.28 ± 3.95	60.27 ± 5.03	9.10 ± 2.19	37.56 ± 4.29	54.75 ± 4.88
L4 U	25.94 ± 2.41	37.59 ± 4.26	54.25 ± 6.56	26.42 ± 2.52	39.77 ± 3.86	57.08 ± 6.08	25.37 ± 2.14	34.96 ± 3.07	50.87 ± 5.37
L4 L		37.36 ± 4.13	55.95 ± 5.97		39.33 ± 3.63	58.57 ± 4.81		35 ± 3.38	52.82 ± 5.69
L4/L5	9.35 ± 2.93	40.57 ± 5.15	59.37 ± 5.5	10 ± 2.97	42.48 ± 5.24	61.49 ± 5.09	8.57 ± 2.68	38.26 ± 3.95	56.81 ± 4.86
L5 U	25.04 ± 2.66	37.34 ± 4.16	56.82 ± 5.87	25.51 ± 2.96	39.2 ± 3.85	59.10 ± 5.27	24.48 ± 2.12	35.12 ± 3.35	54.1 ± 5.38
L5 L		36.45 ± 4.02	55.69 ± 6.68		38.24 ± 3.61	57.76 ± 5.66		34.32 ± 3.38	53.22 ± 6.95

*U* upper margin of disc, *L* lower margin of disc

The most frequent level of aorta division was L4 (58.35%) and the highest level of division found was at the L3/L4 level (2.51%). The aorta diameters (Table 2) were maximal at Th12/L1 levels for all [mean sagittal diameter (mm) ± SD 22.53 ± 3.69; mean coronal diameter (mm) ± SD 22.80 ± 3.67], as well as for gender-separate groups. The minimal sagittal diameter was at the upper margin of L5 (16.62 ± 3.18 mm) and the minimal coronal diameter at the lower margin of L3 (18.14 ± 3.25 mm). Maximal mean sagittal and coronal diameter of the left common iliac artery (LCIA) was at the lower margin of L5 (sagittal 12.05 ± 4.64 mm; coronal: 13.89 ± 5.85 mm) as well as the maximal mean sagittal diameter of right common iliac artery (RCIA) (13.10 ± 4.57 mm). The maximal mean coronal diameter for RCIA was at the upper level of L5 (13.15 ± 4.17 mm). For common iliac arteries (CIAs), minimal mean diameters were at the level of L3/L4 (LCIA sagittal 9.9 ± 1.58 mm; coronal 9.56 ± 1.1 mm; RCIA sagittal 10.1 ± 1.36 mm; coronal 10.12 ± 1.31 mm).

**Table 2:** The measurements of aorta (or common iliac artery right/left) two diameters: sagittal and coronal at the level of intervertebral disc (or upper or lower margin of vertebrae)

	Aorta diameter [mean (mm) ± SD]						Left common iliac artery diameter [mean(mm) ± SD]						Right common iliac artery diameter [mean (mm) ± SD]							
	All group		Male group		Female group		All group		Male group		Female group		All group			Male group			Female group	
	Sagittal	Coronal	Sagittal	Coronal	Sagittal	Coronal	Sagittal	Coronal	Sagittal	Coronal	Sagittal	Coronal	Sagittal	Coronal	Sagittal		Coronal	Sagittal		Coronal
TH12/L1	22.53 ± 3.69	22.80 ± 3.67	23.49 ± 3.79	23.81 ± 3.79	21.38 ± 3.2	21.6 ± 3.12	–	–	–	–	–	–	–	–	–		–	–		–
L1 U	22.38 ± 3.79	22.51 ± 4.29	23.34 ± 3.78	23.67 ± 4.72	21.15 ± 3.65	21.13 ± 3.2	–	–	–	–	–	–	–	–	–		–	–		–
L1 L	20.27 ± 3.69	20.58 ± 3.57	21.25 ± 3.58	21.66 ± 3.41	19.1 ± 3.46	19.29 ± 3.32	–	–	–	–	–	–	–	–	–		–	–		–
L1/L2	19.47 ± 3.61	20.17 ± 3.59	20.47 ± 3.59	21.24 ± 3.56	18.27 ± 3.25	18.9 ± 3.19	–	–	–	–	–	–	–	–	–		–	–		–
L2 U	19 ± 3.42	19.38 ± 3.49	20.04 ± 3.42	20.34 ± 3.44	17.75 ± 2.96	18.24 ± 3.18	–	–	–	–	–	–	–	–	–		–	–		–
L2 L	18.41 ± 3.23	18.5 ± 3.26	19.6 ± 3.21	19.65 ± 3.2	17 ± 2.61	17.14 ± 2.77	–	–	–	–	–	–	–	–	–		–	–		–
L2/L3	18.4 ± 3.28	18.5 ± 3.71	19.59 ± 3.36	19.71 ± 4.16	16.98 ± 2.55	17.06 ± 2.4	–	–	–	–	–	–	–	–	–		–	–		–
L3 U	18.16 ± 3.31	18.28 ± 3.22	19.32 ± 3.5	19.43 ± 3.42	16.77 ± 2.42	16.91 ± 2.29	–	–	–	–	–	–	–	–	–		–	–		–
L3 L	17.66 ± 3.32	18.14 ± 3.25	18.90 ± 3.53	19.46 ± 3.32	16.16 ± 2.27	16.53 ± 2.32	–	–	–	–	–	–	–	–	–		–	–		–
L3/L4	17.81 ± 3.32	18.38 ± 3.47	19.02 ± 3.55	19.73 ± 3.63	16.33 ± 2.25	16.73 ± 2.41	9.9 ± 1.58	9.56 ± 1.1	10.83 ± 1.28	10.56 ± 1.43	9.49 ± 1.52	9.12 ± 0.49	10.1 ± 1.36	10.12 ± 1.31	11.27 ± 1.48		11.02 ± 1.27	9.53 ± 0.9		9.73 ± 1.12
L4 U	17.89 ± 7.74	18.62 ± 3.54	19.27 ± 9.89	19.9 ± 3.7	16.17 ± 2.64	17.01 ± 2.53	10.66 ± 1.75	11.25 ± 2.57	11.75 ± 1.65	12.6 ± 2.9	9.87 ± 1.34	10.26 ± 1.7	10.52 ± 1.77	10.86 ± 1.84	11.52 ± 1.71		11.8 ± 1.77	9.79 ± 1.42		10.18 ± 1.56
L4 L	17.36 ± 3.62	19.82 ± 3.9	18.84 ± 3.73	21.45 ± 3.82	15.66 ± 2.58	17.93 ± 3.05	11.33 ± 2.3	11.53 ± 2.45	12.03 ± 2.39	12.19 ± 2.6	10.47 ± 1.84	10.71 ± 1.95	11.17 ± 2.29	12.3 ± 3.73	11.76 ± 2.18		12.94 ± 3.56	10.43 ± 2.21		11.51 ± 3.79
L4/L5	17.19 ± 3.41	20.25 ± 4.27	18.57 ± 3.06	21.88 ± 3.8	15.59 ± 3.07	18.37 ± 4	11.85 ± 4.83	12.03 ± 4.17	12.72 ± 5.81	12.89 ± 5.13	10.8 ± 2.97	10.98 ± 2.15	11.64 ± 2.82	12.93 ± 5.37	12.35 ± 2.76		13.49 ± 4.17	10.8 ± 2.65		12.26 ± 6.46
L5 U	16.62 ± 3.18	20.93 ± 3.72	17.25 ± 3.25	22.23 ± 3.33	15.42 ± 2.66	18.49 ± 3.14	11.97 ± 3.12	12.4 ± 3.39	12.8 ± 3.38	13.15 ± 3.50	11.0 ± 2.45	11.52 ± 3.02	11.81 ± 3	13.15 ± 4.17	12.66 ± 3.10		13.71 ± 3.97	10.81 ± 2.5		12.48 ± 4.32
L5 L	–	–	–	–	–	–	12.05 ± 4.64	13.89 ± 5.85	12.81 ± 3.96	15.06 ± 6.07	11.14 ± 5.21	12.49 ± 5.24	13.10 ± 4.57	13.04 ± 5.36	13.85 ± 4.97		14.10 ± 5.66	12.18 ± 3.84		11.73 ± 4.65

*U* upper margin of disc, *L* lower margin of disc

The location of the aorta according to its relation to the spinal column (Tables 3 and 4) changes progressively from left (3A, 4A) to the right (1A, 2A) to achieve 2A in 25.23% at the lower margin of L4. However, the dominant location was in 3A at all levels (93.14% at Th12/L1 and lower margin of L1 to 55.45% at L4/L5). The centric aorta location (2½A) extended from 3.97% at Th12/L1 to 22.73% at L4/L5. The location of arterial vessels was similar in the male and female groups with one significant difference at the L3/L4 level (*p* = 0.0133).

**Table 3:** The percentage location of aorta (or common iliac artery left/right) in particular position (1, ½, 2, 2 ½…) at the level of intervertebral disc or upper or lower margin of vertebrae

	Aorta (%)							Left common iliac artery (%)							Right common iliac artery (%)						
	A1	A1 1/2	A2	A2 1/2	A3	A3 1/2	A4	A1	A1 1/2	A2	A2 1/2	A3	A3 1/2	A4	A1	A1 1/2	A2	A2 1/2	A3	A3 1/2	A4
TH12/L1	0	0	2.53	3.97	93.14	0.36	0	0	0	0	0	0	0	0	0	0	0	0	0	0	0
L1 U	0	0	2.18	4.18	92.91	0.18	0.55	0	0	0	0	0	0	0	0	0	0	0	0	0	0
L1 L	0	0	2.17	4.69	93.14	0	0	0	0	0	0	0	0	0	0	0	0	0	0	0	0
L1/L2	0	0	1.99	5.96	91.88	0.18	0	0	0	0	0	0	0	0	0	0	0	0	0	0	0
L2 U	0	0	2.52	7.21	90.09	0.18	0	0	0	0	0	0	0	0	0	0	0	0	0	0	0
L2 L	0	0	8.11	8.47	83.24	0.18	0	0	0	0	0	0	0	0	0	0	0	0	0	0	0
L2/L3	0	0	10.45	10.09	79.46	0	0	0	0	0	0	0	0	0	0	0	0	0	0	0	0
L3 U	0	0	9.71	12.23	78.06	0	0	0	0	0	0	0	0	0	0	0	0	0	0	0	0
L3 L	0	0	15.34	13	71.66	0	0	0	0	0	0	0	0	0	0	0	0	0	0	0	0
L3/L4	0	0	15.87	14.39	69.56	0	0.18	0	0	0	0	100	0	0	0	0	50	20	30	0	0
L4 U	0	0	15.1	16.27	68.63	0	0	0	0	2.22	2.22	95.56	0	0	0	0	60	24.44	15.56	0	0
L4 L	0	0	25.23	16.51	58.26	0	0	0	0	2.39	4.18	89.25	1.49	2.69	0.3	0.6	76.49	9.52	13.1	0	0
L4/L5	0	0	21.82	22.73	55.45	0	0	0	0	5.69	1.82	84.74	2.51	5.24	1.6	0.92	81.92	4.58	10.98	0	0
L5 U	0	0	19.23	11.54	69.23	0	0	0	0.2	9.47	4.55	73.52	2.77	9.47	4	1.2	78.6	4.8	10.8	0.2	0.4
L5 L	0	0	0	0	0	0	0	0	0	0.42	1.67	49.8	8.54	39.58	19.87	7.32	71.34	0.21	1.26	0	0

*U* upper margin of disc, *L* lower margin of disc

**Table 4:** The percentage location of aorta (or common iliac artery left/right) in particular position (1, ½, 2, 2 ½…) at the level of intervertebral disc or upper or lower margin of vertebrae in male and female groups

	Aorta (%)								Left common iliac artery (%)						Right common iliac artery (%)						
	A1	A1 1/2	A2	A2 1/2	A3	A3 1/2	A4	A1	A1 1/2	A2	A2 1/2	A3	A3 1/2	A4	A1	A1 1/2	A2	A2 1/2	A3	A3 1/2	A4
**Male**																					
TH12/L1	0	0	2.31	3.3	93.73	0.66	0	0	0	0	0	0	0	0	0	0	0	0	0	0	0
L1 U	0	0	1.67	3.68	93.31	0.33	1	0	0	0	0	0	0	0	0	0	0	0	0	0	0
L1 L	0	0	2.99	4.32	92.69	0	0	0	0	0	0	0	0	0	0	0	0	0	0	0	0
L1/L2	0	0	1.66	4.98	93.36	0	0	0	0	0	0	0	0	0	0	0	0	0	0	0	0
L2 U	0	0	2.32	6.62	91.06	0	0	0	0	0	0	0	0	0	0	0	0	0	0	0	0
L2 L	0	0	7.95	8.94	83.11	0	0	0	0	0	0	0	0	0	0	0	0	0	0	0	0
L2/L3	0	0	9.6	10.26	80.13	0	0	0	0	0	0	0	0	0	0	0	0	0	0	0	0
L3 U	0	0	9.9	11.22	78.88	0	0	0	0	0	0	0	0	0	0	0	0	0	0	0	0
L3 L	0	0	19.14	14.85	66.01	0	0	0	0	0	0	0	0	0	0	0	0	0	0	0	0
L3/L4	0	0	18.12	16.78	64.77	0	0.34	0	0	0	0	100	0	0	0	0	66.67	33.33	0	0	0
L4 U	0	0	18.31	18.66	63.03	0	0	0	0	0	5.26	94.74	0	0	0	0	68.42	31.58	0	0	0
L4 L	0	0	28.21	15.38	56.41	0	0	0	0	2.7	6.49	89.19	1.08	0.54	0	0	80.98	9.78	9.24	0	0
L4/L5	0	0	23.73	22.03	54.24	0	0	0	0	8.33	2.5	86.25	1.67	1.25	1.26	1.26	81.93	4.62	10.92	0	0
L5 U	0	0	17.65	5.88	76.47	0	0	0	0.37	11.72	4.76	76.56	1.83	4.76	2.56	1.47	80.22	4.4	10.99	0	0.37
L5 L	0	0	0	0	0	0	0	0	0	0.76	0.38	60.84	8.75	29.28	11.41	7.98	78.71	0	1.9	0	0

	Aorta (%)							Left common iliac artery (%)							Right common iliac artery (%)						
	A1	A1 1/2	A2	A2 1/2	A3	A3 1/2	A4	A1	A1 1/2	A2	A2 1/2	A3	A3 1/2	A4	A1	A1 1/2	A2	A2 1/2	A3	A3 1/2	A4
**Female**																					
TH12/L1	0	0	2.79	4.78	92.43	0	0	0	0	0	0	0	0	0	0	0	0	0	0	0	0
L1 U	0	0	2.79	4.78	92.43	0	0	0	0	0	0	0	0	0	0	0	0	0	0	0	0
L1 L	0	0	1.19	5.14	93.68	0	0	0	0	0	0	0	0	0	0	0	0	0	0	0	0
L1/L2	0	0	2.37	7.11	90.12	0.4	0	0	0	0	0	0	0	0	0	0	0	0	0	0	0
L2 U	0	0	2.77	7.91	88.93	0.4	0	0	0	0	0	0	0	0	0	0	0	0	0	0	0
L2 L	0	0	8.3	7.91	83.4	0.4	0	0	0	0	0	0	0	0	0	0	0	0	0	0	0
L2/L3	0	0	11.46	9.88	78.66	0	0	0	0	0	0	0	0	0	0	0	0	0	0	0	0
L3 U	0	0	9.09	13.83	77.08	0	0	0	0	0	0	0	0	0	0	0	0	0	0	0	0
L3 L	0	0	10.76	10.76	78.49	0	0	0	0	0	0	0	0	0	0	0	0	0	0	0	0
L3/L4	0	0	12.7	11.89	75.41	0	0	0	0	0	0	100	0	0	0	0	42.86	28.57	28.57	0	0
L4 U	0	0	11.06	13.27	75.66	0	0	0	0	0	0	100	0	0	0	0	53.85	19.23	26.92	0	0
L4 L	0	0	21.78	17.82	60.4	0	0	0	0	2	1.33	89.33	2	5.33	0.66	1.32	70.86	9.27	17.88	0	0
L4/L5	0	0	19.61	23.53	56.86	0	0	0	0	2.51	1.01	82.91	3.52	10.05	2.01	0.5	81.91	4.52	11.06	0	0
L5 U	0	0	22.22	22.22	55.56	0	0	0	0	6.87	4.29	69.96	3.86	15.02	5.73	0.88	76.65	5.29	10.57	0.44	0.44
L5 L	0	0	0	0	0	0	0	0	0	0	3.23	36.41	8.29	52.07	30.23	6.51	62.33	0.47	0.47	0	0

*A* area, *U* upper margin of disc, *L* lower margin of disc

LCIA was mostly located in 3A (from 49.8% at the lower margin of L5 to 100% at L3/L4). The centric location (2½A) was between 1.67% at the lower margin and 4.55% at the upper margin of L5. 2A was the most common for RCIA (between 50% at L3/L4 and 81.92% at L4/L5). Centric location was from 0.21% at the lower margin L5 to 24.44% at the upper margin of L4.

The most valuable results were those about mean ADD (Table 5). ADD was the shortest at the Th12/L1 level [mean ADD (mm) ± SD 0.90 ± 1.56] and the longest at L4/L5 (2.37 ± 2.89 mm). The same observations were found in male and female groups separately (the shortest ADD in males 0.90 ± 1.50 mm; female 0.91 ± 1.54 mm and the longest ADD in males: 2.69 ± 3.03 mm; females 2.00 ± 2.7 mm). There were statistically significant differences between those groups (males vs. females) at three levels: L1/L2 (1.32 ± 1.97 vs. 0.96 ± 1.78 mm; *p* = 0.0194), L2/L3 (1.97 ± 2.16 vs. 1.15 ± 2.01 mm; *p* = 0.0000), L3/L4 (2.54 ± 2.78 vs. 1.71 ± 2.61 mm; *p* = 0.0012). Distances between discs and CIAs increased from th12/L1 to L4/L5 (from 1.7 ± 1.44 to 3.17 ± 3.42 mm for LCIA and from 3.74 ± 2.3 to 6.97 ± 4.96 mm for RCIA). Significant differences between distances were found for both (left and right) CIAs in the male and female groups (LCIA 3.64 ± 3.63 vs. 2.6 ± 3.06 mm; *p* = 0.0004 and RCIA 7.96 ± 5.06 vs. 5.8 ± 4.57 mm; *p* < 0.001).

**Table 5:** The mean of the shortest distance between aorta (or common iliac artery; with division between left and right common iliac artery) and intervertebral disc (or upper or lower margin of vertebrae) at the particular level of spine

	Aorta–disc (Vertebrae) distance; [mean (mm) ± SD]			Left common iliac artery- disc (Vertebrae) distance; [mean (mm) ± SD]			Right common iliac artery-disc (Vertebrae) distance; [mean (mm) ± SD]		
	All group	Male group	Female group	All group	Male group	Female group	All group	Male group	Female group
TH12/L1	0.90 ± 1.56	0.90 ± 1.50	0.91 ± 1.54	–	–	–	–	–	–
L1 U	2.53 ± 2.04	2.42 ± 2.15	2.72 ± 2.00	–	–	–	–	–	–
L1 L	3.20 ± 2.22	3.33 ± 2.15	2.98 ± 2.05	–	–	–	–	–	–
L1/L2	1.16 ± 1.83	1.32 ± 1.97	0.96 ± 1.78	–	–	–	–	–	–
L2 U	3.07 ± 2	3.09 ± 2.05	3.05 ± 1.92	–	–	–	–	–	–
L2 L	3.76 ± 2.29	4.05 ± 2.54	3.4 ± 1.89	–	–	–	–	–	–
L2/L3	1.6 ± 1.95	1.97 ± 2.16	1.15 ± 2.01	–	–	–	–	–	–
L3 U	3.66 ± 2.77	3.84 ± 2.34	3.32 ± 2.56	–	–	–	–	–	–
L3 L	4.65 ± 2.77	4.92 ± 2.86	4.26 ± 2.4	–	–	–	–	–	–
L3/L4	2.12 ± 2.57	2.54 ± 2.78	1.71 ± 2.61	1.7 ± 1.44	2.56 ± 1.52	1.33 ± 1.23	3.74 ± 2.3	6.16 ± 1.46	2.7 ± 1.74
L4 U	3.5 ± 2.79	3.7 ± 2.96	3.24 ± 2.53	3.17 ± 1.9	3.39 ± 1.98	3.01 ± 1.82	5.98 ± 3.2	7.18 ± 3.01	5.1 ± 3.04
L4 L	4.5 ± 3.66	5.01 ± 4.18	3.91 ± 2.84	5.08 ± 4.57	5.61 ± 5.41	4.43 ± 3.11	8.81 ± 4.93	9.69 ± 5.25	7.74 ± 4.26
L4/L5	2.37 ± 2.89	2.69 ± 3.03	2 ± 2.7	3.17 ± 3.42	3.64 ± 3.63	2.6 ± 3.06	6.97 ± 4.96	7.96 ± 5.06	5.8 ± 4.57
L5 U	3.85 ± 2.76	4.66 ± 2.92	2.22 ± 1.37	6.03 ± 5	6.43 ± 5.6	5.57 ± 4.14	9.37 ± 5.43	9.84 ± 5.41	8.81 ± 5.4
L5 L	–	–	–	10.50 ± 5.01	10.85 ± 4.97	10.08 ± 5.02	10.65 ± 6.28	11.04 ± 5.93	10.18 ± 6.65

*U* upper margin of disc, *L* lower margin of disc

The mean AVD [mean AVD (mm) ± SD] extended from 2.53 ± 2.04 mm at the upper margin of L1 to 4.65 ± 2.77 mm at the lower margin of L3. For males, the maximal mean AVD was at the lower margin of L4 (5.01 ± 4.18 mm) and minimal AVD at upper L1 (2.42 ± 2.15 mm). Maximal AVD for females was at lower margin of L3 (4.26 ± 2.4 mm), minimal at upper L5 (2.22 ± 1.37 mm).

Distances between vertebrae and common iliac arteries increased from upper margin of L4 to lower L5 (from 3.17 ± 1.9 to 10.50 ± 5.01 mm for LCIA and from 5.98 ± 3.2 to 10.65 ± 6.28 mm for RCIA).

## Discussion

There are a lot of studies discussing vascular injury in spine surgery. Vascular complications can occur with an incidence of 1–5 in 10,000 disc operations, they most commonly appear at the L4/L5 level, result in arteriovenous fistulas and predominantly concern injury of common iliac artery, aorta or lumbar arteries [5, 22]. Majority of works discuss that problem only in anterior approach procedures [5, 7, 9, 15, 18, 31]. Nowadays, the posterior approach is considered to minimalize the chance of vascular injury, but it still does not eliminate it—more often it provides long-term complications which can be challenging to diagnose [14, 19, 20, 23]. The requirement of preoperative planning based on CT or magnetic resonance imaging is not debatable; however, there are still scarce studies concerning the anatomical relations between vessels and approach while performing posterior discectomy. Although there are studies indicating the essentiality of such analyses, they mainly highlight importance of surgery based on the anterior approach to the L4/L5 level [7, 15, 18, 31]. Those works are mainly analyzing the levels of bifurcation of the aorta, iliac veins, confluence with the inferior vena cava (IVC), and relations between those structures. What’s more, veins not only occur in great variability in lumbar location [4, 6, 7, 16], but also the measurements of venous structures are imprecise because of variations in blood flow and diameter associated with respiration [11]. That is why we decided to base our study on the relation between spinal column and arterial vessels. We not only did focus on the distances between arterial vessels on spinal vertebrae or discs, but also on the measurements of those anatomical structures and the differences between genders.

Disc measurements are important for the design of artificial intervertebral discs. In our study, the morphometric parameters of IVDs increased consecutively with disc levels (from Th12/L1 to L4/L5) with one exception —the mean height of IVDs at L4/L5 level was smaller than at L3/L4 for all patients and for female only group. Some authors demonstrate that morphometric descriptions are essential to ensure good prosthesis–vertebra contact, better load distribution, and can improve spinal biomechanics [29]. The measurements of vertebrae can be helpful while choosing a suitable length of the surgical instrument during the procedure as the prevention from exceeding the intervertebral space during the posterior approach resulting in injury of vessels. The size of a particular vertebra was in every case smaller than the adjacent discs. We consider these results as indicators for a safe range of operating while removing discs from the intervertebral space. Some authors focused on applying their work directly in clinical proceeding—they established safety work zones to avoid damaging vessels or neural structures [13]. Latest review has elevated that surgical margin for depth of disc space penetration should be considered and kept maximally to 3.0 cm as 5% of discs have diameter as small as 3.3 cm [3]. Other authors have reported that at L4/L5 levels, the more anterior was the position of the nerve root and the more posterior was the position of the retroperitoneal vessels, the more significantly reduced was the safe zone in comparison to upper levels, increasing the risk of nerve and vascular damage [13, 26]. The safe corridor in the aforementioned studies narrows from L1–L2 to the L4–L5 level and is further reduced with rotatory deformity of the spine [26]. The measurements in our work were higher in the male group considering all IVDs and vertebrae-related parameters. Other studies found similar relations with both—gender and lower lumbar geometry[29]. We also did not include the L5/S1 level in our study as the arterial vessels do not adhere to the spinal column at such a low level; therefore, the risk of accidental injury is scarce. Aorta is usually divided higher—only 2.5% divisions occur at the L5/S1 level [4]. What’s more, our analysis showed that CIAs are branching from the spinal structures in more than 5 mm distance in every case at L5 lower margin, what indicates a reduction in the risk of vessel damage from the posterior approach at the L5/S1. Previous studies confirmed our assumption—they showed that the common iliac vessels were closer to the anterior aspect of the intervertebral disc at the L4–L5 as compared with L5–S1 [11, 30]. Achieved results show that common iliac arteries at L5/S1 were within 5 mm of the anterior aspect of the disc space in 23% in women and 19% in men, indicated a significantly increased risk of vessel injury at the L4–L5 level (respectively, 66% of the common iliac arteries in women and 49% of those in men were within 5 mm of the anterior aspect of the disc space) [11]. The aorta bifurcation in our study group appeared in the majority at L4 level, which is consistent with CT-based study of Datta et al. [9] or anatomical results of Aschini et al. who reported the similar results [4]. Cadaver study by Panagouli et al. [21] also confirmed our findings—mean level of bifurcation was the lower third of the L4 vertebral body. As aforementioned, there is variability of venous structures in the lumbar region that lead to discrepancies in different studies [4, 6, 7, 15, 16]. Some authors reported aorta bifurcation 1–2 segments above the IVC confluence [15]. On the other hand, we established that IVC confluence is located around the body of L4 in 80% of cases [4]. Reported differences of IVC confluence location at L4 can be explained by the different range of age and shortening of the spinal column through that, which result in lower placement of the bifurcation of aorta [4]. Furthermore, there are other factors potentially influencing the level of aorta, such as lumbarization, sacralization, and lumbar lordosis angle [4]. Study by Berger et al.[2] has found that age itself do not influence location of aortic bifurcation, but patients who were both elderly and thinner had lower aortic bifurcation and a trend towards a lower IVC bifurcation. What’s more, we analyzed the course of the aorta in relation to the spinal column for a better description of the prevertebral vessels. At all levels, the aorta was located in A3 of our classification, what implies that its course is generally left-sided, with no difference in gender. In the lowest lumbar levels, in a similar number of cases, it was investigated centrally or to the right side. The distances between vessels and disc at the following levels L1/L2, L2/L3, L3/L4 were significantly smaller in females. Similar relationship was found for both CIAs. ADD was the shortest at the Th12/L1 level and the longest at L4/L5. Analogous observation was in male and female groups separately. That indicates a higher risk of vessel injury at higher levels of the spine, where the aorta even adheres to discs with no separating space. Furthermore, females are at higher risk of such complications when discectomies are performed considering less space between discs and prevertebral vessels. Although we did not investigate influence of age on spine morphometrics, Shao et al. [28] have shown that heights of lumbar discs increase, while concavity index decreases linearly with age. Other study found that longitudinal diameters of lumbar intervertebral foramina decrease with age [8]. In aging intervertebral disc, intervertebral chondrosis and intervertebral osteochondrosis take place. These processes are combined with typical dislocations of intervertebral disc tissue in an anterior or dorsolateral direction [25]. Radiographic study performed by Garg et al. [12] has recommended that preoperative analysis of morphometrics would be useful especially in lateral lumbar interbody fusions at L4–L5 levels and in females. Their research has shown that morphometric parameters differ between males and females, and patients’ sex may affect safe working zones in spinal procedures. We have been assessing patients without great deformities of spine, but influence of degenerative lumbar spine disease (DLSD) in study population has not been established. Abbas et al. have shown that degenerative lumbar spine stenosis affects its morphometrics. In their study, vertebral body length and width were greater in stenosis group in comparison to general population. As our study aimed to mimic general population, influence of DLSD may be omitted. In our study, patients were examined in a prone position. Some authors suggest discrepancies between vessel situation and the prone or supine position of the patient [30]. There is a report that the aortic bifurcation and confluence of the common iliac veins were most commonly at the level of the L4 vertebral body and migrated cranially with prone positioning. It was also associated with greater distances between the disc and iliac vessels at L4–L5 and L5–S1 by an average of 3 mm [30]. Others showed little change in these measurements between different positions and established that the use of bolsters to decompress the abdominal contents in the prone position did not significantly alter ADD [11].

Our work is the morphometric description of the spine and prevertebral vessels of the lumbar region that can be used during preoperative planning as a suggestion concerning the surgical approach, size of instruments or spinal implants. Surgeons should be aware of potential differences between genders, although, those results can be interfered by such factors as patient’s height or weight. Surgical safety during removal of intervertebral disc is a key. We propose to elevate protection by measuring discs length and width in all cases and in cases without spine morphometrics analysis, we recommend adhering to standard boundaries of 2.5–3 cm for devices and instruments that are used in intervertebral space. Special awareness should be applied when L3/4 and L4/5 discs are dissected as at those levels division of aorta may take place and area in which large vessels anatomy is the most variable.

Although, many previous works discussed limited disc levels, there is lack of works which take into consideration all lumbar regions and anatomical relations among discs, vertebrae, and prevertebral vessels [6, 7, 15, 18, 31]. Our work is, to our knowledge, one of the vastest analyses of such a matter.

## Data Availability

No data and materials are going to be available publicly.
